# Cardiac regeneration: Pre-existing cardiomyocyte as the hub of novel signaling pathway

**DOI:** 10.1016/j.gendis.2023.01.031

**Published:** 2023-03-24

**Authors:** Tao Wang, Xinzhe Chen, Kai Wang, Jie Ju, Xue Yu, Wanpeng Yu, Cuiyun Liu, Yin Wang

**Affiliations:** a Institute for Translational Medicine, The Affiliated Hospital of Qingdao University, College of Medicine, Qingdao University, Qingdao, Shandong 266023, China; b College of Medicine, Qingdao University, Qingdao, Shandong 266023, China

**Keywords:** Cardiac regeneration, Cell cycle re-enter, Myocardial infarction, Pre-existing cardiomyocyte proliferation, Regenerative therapy of cardiovascular diseases

## Abstract

In the mammalian heart, cardiomyocytes are forced to withdraw from the cell cycle shortly after birth, limiting the ability of the heart to regenerate and repair. The development of multimodal regulation of cardiac proliferation has verified that pre-existing cardiomyocyte proliferation is an essential driver of cardiac renewal. With the continuous development of genetic lineage tracking technology, it has been revealed that cell cycle activity produces polyploid cardiomyocytes during the embryonic, juvenile, and adult stages of cardiogenesis, but newly formed mononucleated diploid cardiomyocytes also elevated sporadically during myocardial infarction. It implied that adult cardiomyocytes have a weak regenerative capacity under the condition of ischemia injury, which offers hope for the clinical treatment of myocardial infarction. However, the regeneration frequency and source of cardiomyocytes are still low, and the mechanism of regulating cardiomyocyte proliferation remains further explained. It is noteworthy to explore what force triggers endogenous cardiomyocyte proliferation and heart regeneration. Here, we focused on summarizing the recent research progress of emerging endogenous key modulators and crosstalk with other signaling pathways and furnished valuable insights into the internal mechanism of heart regeneration. In addition, myocardial transcription factors, non-coding RNAs, cyclins, and cell cycle-dependent kinases are involved in the multimodal regulation of pre-existing cardiomyocyte proliferation. Ultimately, awakening the myocardial proliferation endogenous modulator and regeneration pathways may be the final battlefield for the regenerative therapy of cardiovascular diseases.

## Introduction

Cardiovascular diseases (CVDs) remain the primary health concern for the world population, accounting for the largest fraction of deaths related to noncommunicable diseases.^1^ Among the least renewable cells in the human body, cardiomyocytes renew approximately 1% annually. Myocardial infarction (MI) is a leading cause of death in CVDs, with a prevalence of more than 5.8 million in the United States and 23 million worldwide, and it is still on the rise.^2^^,^^3^ In developed countries, early mortality rates related to acute coronary heart disease have fallen sharply.^4^ However, the heart damage caused by disease is not easily restored to normal.

**Figure 1:**
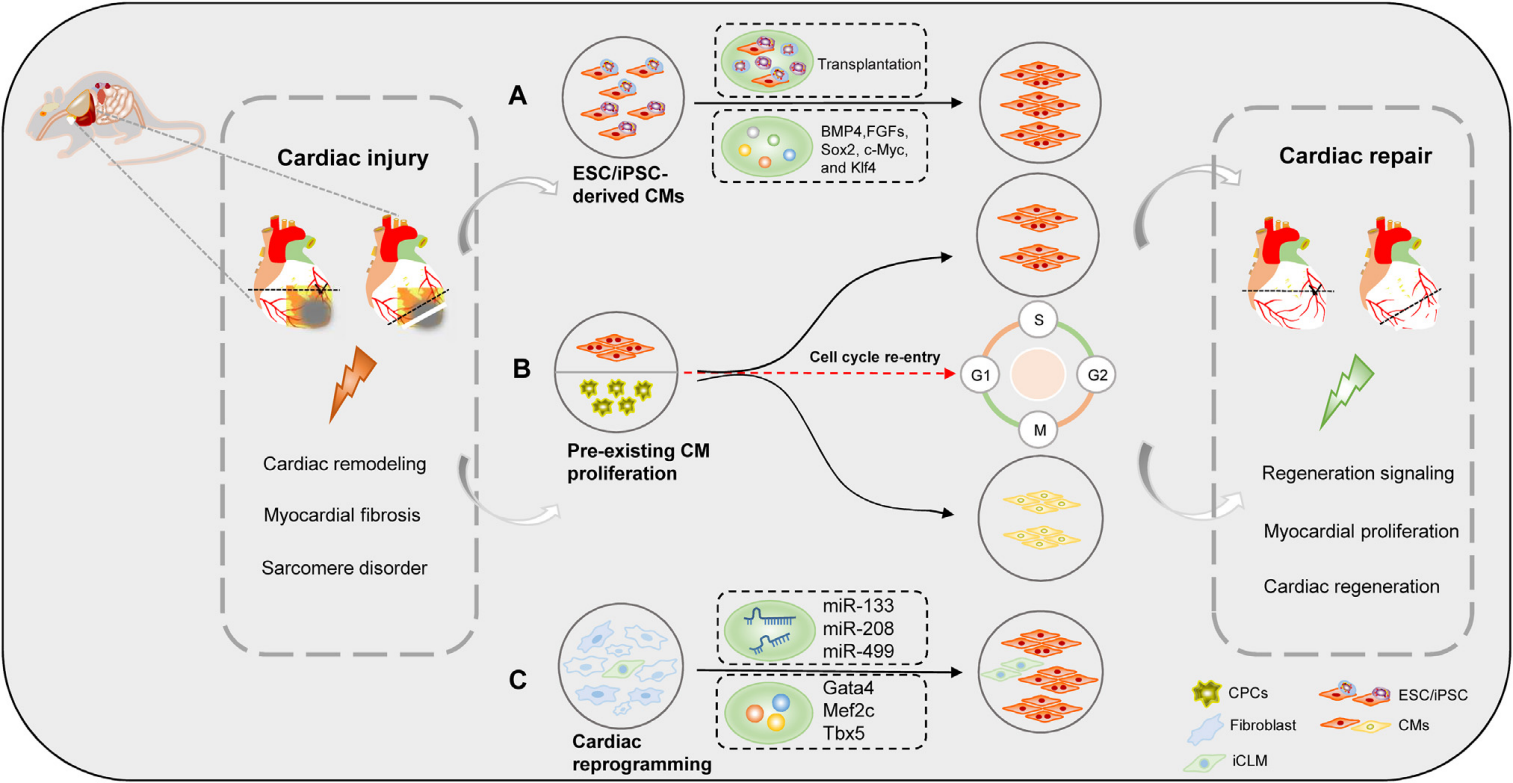
Mainstream therapeutic strategies for cardiac regeneration and repair. **(A)** The cardiac-specific transcription factor plays an important role in the regulation of cardiac differentiation from embryonic stem cells (ESCs), including BMP4, FGFs, Sox2, Myc, and Klf4. **(B)** Pre-existing cardiomyocyte (CM) proliferation triggers endogenous regeneration. Multiple key regulators drive cardiomyocyte cycle entry and trigger CM proliferation. **(C)** The transcription factors (GATA4, HAND2, and T-box5) and microRNAs (miR-1, miR-133, miR-208, and miR-499) efficiently reprogrammed postnatal cardiac or dermal fibroblasts directly induced cardiac-like myocytes (iCLMs).

Cardiac regeneration is a powerful process involving regenerative medicine by which parts of the body are restored after MI. Embryonic stem cells (ESCs)/human induced pluripotent stem cells-derived cardiomyocytes (iPSC-CMs), cardiac reprogramming, and pre-existing cardiomyocyte proliferation trigger cardiac regeneration in different ways (Fig. 1).^5^, ^6^, ^7^, ^8^, ^9^, ^10^ In recent years, iPSC-CM transplantation and revascularization techniques are powerful treatment strategies for ischemic heart failure. IPSC-CMs have been transplanted into damaged myocardial tissue assisted by bioscaffolds. Compared with autologous transplantation, long-term immunosuppression in allogeneic transplantation results in transient or persistent arrhythmias.^11^ Thus, allogeneic iPSC-CMs need to control the occurrence of arrhythmia.

Notably, pre-existing cardiomyocyte proliferation is one of the key strategies for cardiac regeneration. Genetic lineage tracing analysis has demonstrated the newly formed myocardium is derived from pre-existing cardiomyocytes.^12^ Pre-existing cardiomyocyte proliferation compensates for heart failure caused by loss of functional cardiomyocytes and cardiac remodeling.^13^ However, pre-existing cardiomyocyte proliferation requires extremely complex synergistic effects, including dedifferentiation, proliferation, and re-differentiation.^14^ A variety of complex regulation modes and signal pathways together form crosstalk, which will improve the regeneration capacity of the heart.

In this review, we focus on the application of the cardiac cell cycle activating key modulator axis and the emerging star proteins in cardiac regeneration signaling pathways, such as cell cycle-associated functional proteins (CHK1), low-density lipoprotein receptor-related protein 6 (LRP6), pyruvate kinase muscle isozyme 2 (PKM2), homeobox B13 (HOXB13), and MEIS2. Noncoding RNAs (ncRNAs), such as microRNAs (miRNAs), long noncoding RNAs (lncRNAs), and circular RNAs (circRNAs), have played an increasingly critical role in cardiomyocyte proliferation and cardiac regeneration. Therefore, the way to fundamentally prolong the postnatal window of myocyte proliferation and re-enter the cell cycle is a new challenge for cardiac regeneration and repair.

## Pre-existing cardiomyocytes as a promising strategy for cardiac regeneration

Traditionally, the growth and development of the mammalian heart exist only in the embryonic stage. At that time, myocardial cells can divide and proliferate. When a fetus is born, myocardial cells exit the cell cycle and stop cell proliferation, making it difficult for the heart to develop tumor-related diseases.^13^^,^^15^ While this mechanism has benefits for the human body, it also has potential hazards. When a damaged heart is not able to provide new cells, what can be done to repair the damaged tissue? Studies have shown that additional endogenous cell sources may contribute to the regeneration of the entire ventricle.^13^

The low level of endogenous myocardial proliferation in adult mammals dictates that loss of cardiomyocytes due to cardiac injury makes it difficult to automatically replenish new cells. To promote endogenous cardiomyocyte proliferation, researchers have sought to promote cell cycle progression using combinations of cell cycle regulators, such as cyclins, cyclin-dependent kinases (CDKs), and tumor suppressor genes.^16^ Genetic fate mapping shows that the source of most cardiac regeneration after cardiac injury is pre-existing cardiomyocytes. Recently, Zeng et al revealed that the division of pre-existing cardiomyocytes is the primary origin of new cardiomyocyte formation.^17^ *In vitro*, using heart-specific transgenic mice to track the fate of adult cardiomyocytes, the proliferation rate of mononuclear and binuclear ACMs in co-culture was close to 7.0%.^17^ *In vivo*, an anti-ischemic ligand protein 43 mutant enhanced the formation of new cardiomyocytes in the border zone of myocardial infarction and improved cardiac function. In conclusion, the proliferation of pre-existing cardiomyocytes is a potential source of new cardiomyocyte formation and an effective strategy for cardiac regeneration after cardiac injury.

## Multimodal regulation of pre-existing cardiomyocyte proliferation

**Figure 2:**
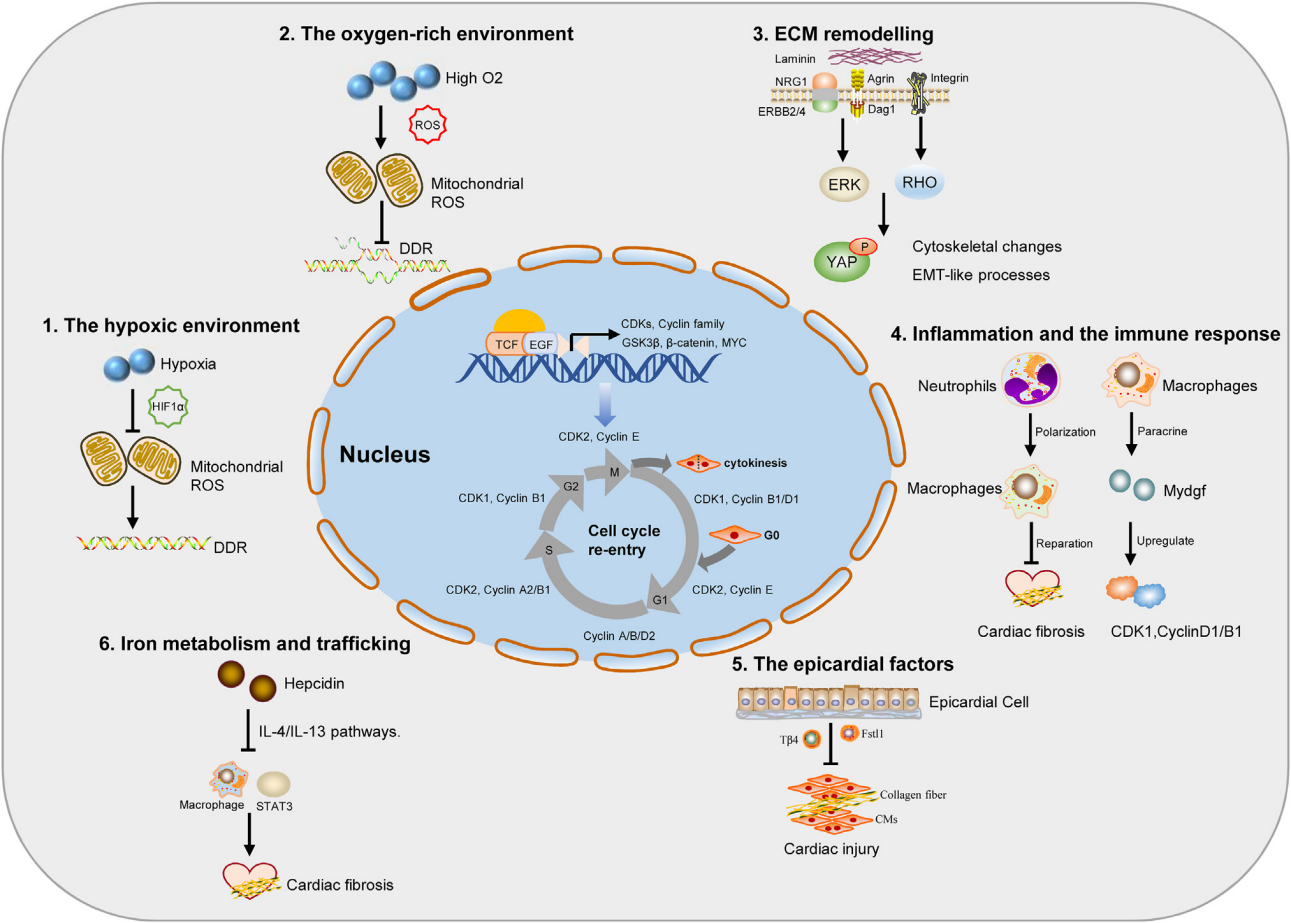
Schematic overview of multimodal regulation for cardiomyocyte (CM) cycle re-entry and myocardial regeneration. According to the above, we summarize the cell cycle-related multimodal regulation factor and CM microenvironment in adult mammalian CM proliferation and heart regeneration. The extracellular matrix (ECM) proteins, reactive oxygen species (ROS), epicardial factors, hypoxic environment, iron metabolism, inflammation, and the immune response awake adult CMs to re-enter the cell cycle and trigger cardiac regeneration and repair.

Previous studies have shown that mammalian hearts seem to have the ability to regenerate during a short period after birth. Cardiomyocyte proliferation during cardiac development appears to be tightly controlled by some specific regulatory networks or factors. Consequently, there is a growing initiative to focus on reactivating the cell cycle of mouse cardiomyocytes to prolong the window of cardiac regeneration. In order to restart cardiomyocyte proliferation in the adult mammalian heart, a comprehensive understanding of the molecules that control cardiomyocyte cycle regulation is required. In recent years, cell cycle triggers have induced the dedifferentiation and proliferation of robust cardiomyocytes and have reactivated the dormant postnatal regeneration window in mice.^18^ Induction of cardiomyocyte proliferation using these different means is an attractive therapeutic option for improving cardiac function and heart failure. In the following section, we summarize the cell cycle-related process and key factors in adult mammalian cardiomyocyte proliferation and heart regeneration (Fig. 2).

## Extracellular matrix remodeling

The extracellular matrix (ECM) plays a critical role in providing the necessary microenvironment for the normal regeneration of heart tissue.^19^ After a heart injury occurs, it causes a continuous and irreversible fibrotic reaction, resulting in cardiac structural disorders and cardiac dysfunction. Myocardial fibrosis is caused by an imbalance in the production and degradation of the ECM, which leads to the formation of scar tissue in the intercellular substance. Nevertheless, the latest research has revealed that some ECM proteins are involved in regulating myocardial proliferation and heart repair.^20^ Type I collagen promotes the proliferation and differentiation of myofibroblasts by reducing the expression of α2β1 integrin in myocardial tissue and then regulating the downstream target genes PTEN and AKT. In addition, type III collagen can effectively activate fibroblast proliferation, while type VI collagen can effectively trigger fibroblast differentiation.^21^

Agrin and its receptor α-dystroglycan serve as important components of ECM.^22^ In adults, agrin treatment promotes cardiomyocyte proliferation via Yes-associated protein (YAP) and extracellular signal-regulated kinases (ERK)-mediated signaling.^23^ In 2020, Kstler et al revealed that a single dose of recombinant human agrin improved cardiac fibrosis and poor remodeling 28 days after MI. ERBB2^24^ receptors are the most significant neuregulin-1 (NRG1)^25^ receptors expressed in embryonic, fetal, and neonatal cardiomyocytes. Transient overexpression of ERBB2 can promote the interaction of YAP with the nuclear membrane and cytoskeleton components and epithelial–mesenchymal transition (EMT)-like regeneration, which is manifested in cytoskeleton remodeling, junction dissolution, migration, and ECM transition.^18^^,^^26^ Neonatal rats were partially digested into fetal cardiac ECM, which released fewer cross-linking components, matured sarcomere, and promoted cardiomyocyte proliferation. The results implied that the adult heart may retain the regeneration clues masked by more abundant and mature ECM components.

## Epicardial factors

To understand why neonatal mice can regenerate their hearts, researchers have focused on epicardial factors related to the cell differentiation of many tissues. Baf60c is a component of the adenosine triphosphate-dependent chromatin remodeling complex, which plays an important role in early cardiac development. Interestingly, the time of baf60c up-regulation was consistent with that of cardiomyocyte proliferation during ventricular resection in neonatal mice.^27^ This is the first study to show that baf60c contributes to heart regeneration in vertebrates. Follistatin-like 1 (FSTL1), as an important activator of epicardial cells, participates in various damage repair effects after MI. Notably, applying the human FSTL1 protein via an epicardial patch promotes the re-entry of the cell cycle and the division of original cardiomyocytes and improves cardiac remodeling and fibrosis.^28^ Taken together, the data indicate that epicardial factor reconstitution regenerates the adult mammalian heart.

## The hypoxic environment

Compared with adult cardiomyocytes, why do neonatal cardiomyocytes have significant proliferation potential? Recently, it has been reported that in mice that are gradually exposed to severe systemic hypoxemia, inhaled oxygen significantly diminished by 1% for 14 days, resulting in the restraint of oxidative metabolism, reactive oxygen species (ROS) levels, and oxidative DNA damage response (DDR).^12^ In the mammalian uterus, endothelial cardiomyocytes proliferate actively to form primitive hearts, but they stop dividing soon after birth. Intriguingly, postnatal hypoxia and low ROS prolonged the postnatal proliferation window of cardiomyocytes, while oxygen enrichment and high ROS production significantly inhibited cardiomyocyte proliferation.^29^

Through bioinformatics and biochemical analysis, hypoxia-inducible factor-1 α (HIF1 α), a TF activated by hypoxia, mediates the proliferative response during heart development.^30^^,^^31^ HIF1α deficiency led to the activation of ATF4 and p53, which reveals the potential mechanism of the HIF1α, ATF4, and p53 oxidative stress response in regulating cardiomyocyte proliferation.^32^ Nuclear connexin novex-3 is the largest protein found in the nucleus of mouse cardiac muscle during the hypoxia fetal period, from the Z-disk, I-band, and A-band to the M-band of the myocardial and skeletal sarcomere.^33^ In an oxygen-deficient environment, novex-3 knockout significantly decreased the proportion of histone H3 phosphorylated at serine 10 (pH3) and Aurora B kinase-positive fetal cardiomyocytes (fCMs).^34^ A recent study shows that fam64a is a new cycle-promoting regulatory molecule. The expression of fam64a and the degradation of the anaphase-promoting complex/cyclostome are necessary for the division of embryonic cardiomyocytes.^35^ These data suggest that novex-3 and fam64a play a role in cell cycle promoters in hypoxia-induced fCMs.

In contrast, the oxygen-rich state induces cardiomyocyte cell-cycle arrest. The levels of ROS and DDR markers gradually increase on the 7th day postnatal or after MI.^29^^,^^36^ However, postnatal hypoxemia, scavenging of ROS, or inhibition of DDR can prolong the proliferation window of postnatal cardiomyocytes.^37^ In the process of MI, Nrf2 was used to activate the expression of paired-like homeodomain 2 (PITX2). Then, PITX2 was combined with YAP, which activated the ROS scavenger and protected the cells from oxidative damage.^38^

## Inflammation and the immune response

Multiple injury models suggest that inflammatory signaling is a trigger to induce cell proliferation or differentiation of progenitor cells to restore lost tissue substrates.^39^ When an inflammatory response occurs in MI, neutrophils and macrophages participate in different stages of infarct healing. A large number of neutrophils infiltrate the infarct area to produce high levels of ROS and secrete protease, which aggravates local vascular and tissue damage.^40^ Subsequently, monocyte-derived macrophages are summoned to the heart and remove debris and apoptotic neutrophils, activating the cardiac regeneration and repair pathway.^41^^,^^42^ Recent studies found that in MI mice, neutropenia deteriorated, fibrosis increased, and heart failure progressively developed. However, the number of macrophages increased, which was related to the decreased mobilization of LY6C^high^ monocytes and the increased proliferation of myocardial macrophages.^43^ A bioinformatics secretome analysis was conducted on the bone marrow cells of patients with acute MI to determine the newly secreted protein with therapeutic potential in promoting the survival of cardiomyocytes and angiogenesis. Bone marrow-derived monocytes and macrophages endogenously produce this protein, bone myeloid-derived growth factor (MYDGF), to protect and repair the heart after MI.^44^ MYDGF controls cardiomyocyte division by activating the c-MYC/FoxM1 pathway, enhances cardiac repair after heart injury in neonatal and adult mice, and provides a potential target for reversing heart remodeling and heart failure.^45^ Toll-like receptors (TLRs) are pattern recognition receptors that play a pivotal role in inducing innate immunity and inflammation.^46^ TLR3 is necessary for the regeneration of damaged hearts in newborns. TLR3 activation induces glycolysis-dependent YAP and targets the cell cycle inhibitor DNMT1/p27, leading to the augmentation of neonatal cardiomyocyte proliferation.^47^ The results showed that the combination of four cell cycle regulators composed of cyclins and CDK complexes could effectively induce cardiomyocyte proliferation and subsequent cell survival *in vitro* and *in vivo*.

## The vital role of iron metabolism and trafficking

Iron overload has an adverse effect on approximately one-third of patients with coronary heart disease and heart failure. Current research has also incorporated systemic and local iron metabolism defects into the focus of cardiovascular medical research. Hepcidin is a major sensor that regulates iron trafficking.^48^^,^^49^ In a model of apical resection-induced heart regeneration in neonatal mice, hepcidin-deficient macrophages promoted cardiomyocyte proliferation. Hepcidin deficiency enhances the number of inflammatory macrophages and inhibits macrophage-induced cardiac repair and regeneration by regulating the interleukin (IL)-4/IL-13 pathway.^50^^,^^51^ The matricellular protein CCN1 promotes the DDR pathways and provokes p53 and the RAC1-NOX1 complex to produce ROS.^52^^,^^53^ This leads to the activation of the ROS-dependent p16INK4a/pRb pathway, which promotes the rapid expression of aging and anti-fibrosis genes. Studies have found that CCN1 induces fibroblast senescence and triggers the expression of senescence-related phenotypic factors *in vitro*, including the expression of IL-1a and IL-6, which in turn promotes the proliferation of neonatal cardiomyocytes.^54^

## Signaling pathways in pre-existing cardiomyocyte proliferation

### Hippo pathway

The Hippo-YAP pathway is a newly established signaling pathway that mitigates cell growth and organ size.^55^^,^^56^ The Hippo kinase, the *Drosophila* homolog of the mammalian Ste20-like kinases (MST1) and MST2, promotes proper termination of cell proliferation and restrain heart size during development.^57^, ^58^, ^59^ Characterization and mutation analysis of the mammalian core kinase cascade revealed the conservation of the Hippo pathway and its role in mammalian organ size regulation.^60^^,^^61^ The core components of the Hippo pathway are kinase cascade, including MST1, MST2, LATS1, LATS2, the adaptor proteins Salvador 1, and MOB kinase activators 1A and MOB1B, the homologous transcriptional co-activator YAP, transcriptional co-activator with PDZ-binding motif (TAZ), and the TEAD.^62^^,^^63^ In mammals, once the extracellular growth inhibition signal is sensed, a series of kinase cascades of phosphorylation reactions are activated, phosphorylation of downstream effect factors YAP and TAZ, which makes YAP stay in the cytoplasm and reduces its nuclear activity, thus achieving the regulation of cell cycle and organ size.^64^^,^^65^

Recent studies have shown that the Hippo pathway is up-regulated in human MI, while the Hippo pathway closure is reversed in mice, which increases scar border vascularity, reduced fibrosis, and recovery of pumping function.^55^ As mentioned previously, YAP expression was robustly detected in fetal, neonatal, and adult mouse hearts and declined with age. Through a YAP5SA gain-of-function transgenic mouse, cardiomyocytes acquire a more primitive cell state, and mononuclear diploid cardiomyocytes are enriched and increase chromatin accessibility at TEAD elements in YAP5SA hearts.^65^ In addition, together with the Hippo pathway, mechanical signal transduction regulates YAP activity in an integrated way. YAP and TAZ are considered conservative mechanical sensors. Cells convert these stimulating signals into biochemical signals that control many aspects of cell behavior, including cell growth, differentiation, and cancer progression. Activation of YAP requires downstream signal pathways of ERBB2, including reconstituted cytoskeleton and nuclear envelope components, and ERK-dependent mitotic phosphorylation of YAP to S274 and S352.^18^ In general, many studies have revealed that the Hippo-YAP pathway plays a key role in regulating heart development, growth, and homeostasis. YAP/TAZ, as a potential regulator of tissue and organ regeneration, is a promising therapeutic target for cardiac regenerative medicine.

### Notch signaling pathway

As first proposed by Morgan, the Notch signaling pathway has been shown to play a pivotal role in the regulation of many fundamental cellular processes, including cell fate determination, development, differentiation, tissue homeostasis, apoptosis, and regeneration.^66^^,^^67^ Previous studies have shown that partial amputation of the zebrafish ventricle significantly increased expression of the Notch signaling components Notch1b and delta C.^68^ The components of the Notch signaling pathway were gradually up-regulated with cardiac regeneration, suggesting that this pathway may play a key role in mammalian embryonic and adult cardiogenesis processes.^67^ During embryonic and adult heart development, Notch expression and signaling decline gradually. Transient overexpression of exogenous activated Notch intracellular domain augments proliferative signaling in infarcted myocardium, as an important mediator of cardiac repair and regeneration, which ameliorates heart failure four weeks after treatment.^14^^,^^69^ Nevertheless, silencing of Notch in embryonic cardiomyocytes results in atrial and ventricular septal defects.^70^ Studies indicate that Notch receptors are enhanced in endocardial and epicardial cells after apical amputation.^68^ Notch signaling is necessary for epicardial activation and cardiac regeneration. Unexpectedly, hyperactivation of Notch signaling also suppressed cardiomyocyte proliferation and the sensitivity of regeneratives, illustrating the crosstalk between Notch and the other signaling hubs during regeneration.^67^, ^68^, ^69^, ^70^, ^71^, ^72^

In addition, one study showed Notch signaling activation to be mediated by hemodynamic alteration after injury. In 2021, researchers first demonstrated that endocardial primary cilia mediated the up-regulation of Krüppel-like factor 2 gene expression, which is a newly emerging field of mechanical stress signal in regulating the Notch signaling pathway and myocardial regeneration.^73^ For these reasons, Notch signaling is a highly conserved pathway during fetal and adult heart development that diminished the percentage of heart scars, ameliorated cardiac function, and enhanced cardiomyocyte proliferation.

### Wnt/β-catenin signaling pathway

Since the initial discovery of the first Wnt1 gene in 1982 by Nusse and Varmus, interest in the Wnt/β-catenin signaling pathway and emerging diseases therapeutic modalities are continuously increasing.^74^^,^^75^ The Wnt/β-catenin signaling pathway has played an increasingly prominent role in progenitor cell fate, embryonic development, homeostasis, and tissue regeneration. The core components of the Wnt/β-catenin signaling pathway include the low-density lipoprotein receptor-related protein (LRP) family, Frizzleds (FZDs) family, the adenomatous polyposis coli (APC) gene, the GSK3β, the scaffold protein Axin, and casein kinase 1, which are involved in regulating cell proliferation and renewal.^76^ When the Wnt pathway is on, the Wnt protein directly binds to the membrane receptor FZD and the auxiliary receptor LRP-5/6, promoting the association of Axin with phosphorylated LRP.^77^ The Axin/APC/GSK3β/β-catenin cytoplasmic protein complexes dissociated and existed stably in the cytoplasm, β-catenin translocates into the nucleus and binds with nuclear t-cell factors (TCF)/lymphoid enhancer-binding factor (LEF), which enhances the transcription and expression of target genes such as c-MYC, cyclin D1, and CDKN1A.^78^^,^^79^

Increasing evidence shows that Wnt/β-catenin signaling is a key trigger of cardiac regeneration, which drives neonatal cardiomyocyte proliferation *in vivo*. In mature cardiomyocytes, N-cadherin and β-catenin are associated with each other to form an intercellular adhesion junction protein complex, thereby restraining the secretion and translocation of β-catenin. The treatment of N-cadherin antibody and CHIR99021 GSK inhibitor augments the release of β-catenin and nuclear translocation, which leads to the reactivation of the Wnt pathway and adult myocardium.^80^ In particular, constitutively active β-catenin distinct transcriptional networks form a proliferative effect to a cardioprotective effect in adult mice post-MI.^81^ Hopefully, the Wnt/β-catenin signaling pathway will create a new field based on tissue engineering and molecular biology to transform clinical regenerative medicine, which will provide significant value in cardiovascular treatment.

## Emerging non-classical signaling pathways

### NcRNAs network in pre-existing cardiomyocyte proliferation

In the past few decades, ncRNA has gained intensive research in the biomedical field, which is emerging as a new fundamental regulator of gene expression.^82^, ^83^, ^84^, ^85^ NcRNAs, such as miRNAs, lncRNAs, and circRNAs, have been reported to play critical roles in the regulation of cardiac regeneration and cardiomyocyte physiologic and pathologic signaling.^86^^,^^87^ Accordingly, elucidating the molecular mechanisms and signaling pathways of ncRNAs regulating the pre-existing cardiomyocyte cycle is the key to advancing cardiac regeneration therapy.^88^ In Table 1, we summarize ncRNAs that play an important role in cardiomyocyte proliferation and their regulatory targets.

**Table 1:** Multiple ncRNAs trigger pre-existing cardiomyocyte proliferation signaling pathways.

Modulator	Cardiomyocyte proliferation	Target of action	Molecular mechanism of interaction
*miRNA*			
miR-152^47^	Up-regulation	p27, DNMT1	miR-152 represses P27kip1/DNMT1 expression, leading to cardiomyocyte proliferation.
miR-1825^89^	Up-regulation	NDUFA10	miR-1825 inhibits NDUFA10 expression and promotes cardiomyocyte proliferation.
miR-199a-3p^90^	Up-regulation	Cd151	miR-199a-3p promotes cardiomyocyte proliferation by binding to Cd151 and inhibiting Mapk11 expression.
miR-19a/19b^91^	Up-regulation	cyclin B1, cyclin D1, and CDK1	miR-19a/19b up-regulate expression of cell cycle-related proteins, cyclin B1, cyclin D1, and CDK1, promotes cardiomyocyte proliferation.
miR-204^92^	Up-regulation	Jarid2	miR-204 activates Cyclin A, Cyclin B, cyclin D2, and PCNA levels, and promotes cardiomyocyte proliferation.
miR-294^93^	Up-regulation	Wee1	miR-294 represses Wee1 expression, increases cyclin B1/CDK1 complex activity, and promotes cardiomyocyte proliferation.
miR-25^94^	Up-regulation	FBXW7	miR-25 represses FBXW7 expression, increases PCNA expression, and promotes cardiomyocyte proliferation.
miR-31a-5p^95^	Up-regulation	RhoBTB1	miR-31a-5p represses RhoBTB1 expression and increases cardiomyocyte proliferation.
miR-301a^96^	Up-regulation	PTEN	miR-301a represses PTEN/AKT signaling pathway and increases cardiomyocyte proliferation.
miRNA-1^97^	Down-regulation	IGF1, cyclin D1	miRNA-1 directly suppresses the cyclin D1 and decreases cardiomyocyte proliferation.
miRNA-128^98^	Down-regulation	SUZ12	miRNA-128 reduces Cyclin E and CDK2 activity by inhibiting SUZ12 expression and arrests cardiomyocyte proliferation.
miRNA-127-3p^99^	Down-regulation	KMT5A	miRNA-127-3p arrests cardiomyocyte proliferation through inhibition of KMT5a.
miRNA-29a^100^	Down-regulation	cyclin D2	miRNA-29a inhibits the expression of cyclin D2 and arrests cardiomyocyte proliferation.
miRlet-7i-5p^101^	Down-regulation	cyclin D2, E2F2	miRlet-7i-5p inhibits the expression of cyclin D2, E2F2 and arrests cardiomyocyte proliferation.
*LncRNA*			
Sirt1 Antisense^102^	Up-regulation	Sirt1	Sirt1 antisense lnc RNA binds to the Sirt1 3′-untranslated region, enhances the stability of Sirt1, increases Sirt1 abundance, and up-regulate cardiomyocyte proliferation.
NR_045363^103^	Up-regulation	miR-216a	NR_045363 sponge adsorbes miR-216a, blocking the inhibition of miR-216a on JAK2, activates the JAK2-STAT3 pathway and promotes cardiomyocyte proliferation.
ECRAR^104^	Up-regulation	ERK1/2	ECRAR activates E2F1-ECRAR-ERK1/2 signaling and promotes cardiomyocyte proliferation.
CPR^105^	Down-regulation	DNMT3A, MCM3	CPR directly interacts and recruits DNMT3A to MCM3 promoter cysteine-phosphate-guanine sites, silences MCM3, and suppresses cardiomyocyte proliferation.
CRRL^106^	Down-regulation	miR-199a-3p	CRRL binds to miR-199a-3p, increases the expression of Hopx, and arrests cardiomyocyte proliferation.
CAREL^107^	Down-regulation	miR-296	CAREL inhibits miR-296 expression, leading to up-regulation of Trp53inp1 and Itm2a and arrests cardiomyocyte proliferation.
*CircRNA*			
HiPK3^108^	Up-regulation	N1ICD	HiPK3 increases the acetylation and stability of N1ICD and promotes cardiomyocyte proliferation.
Nfix^109^	Down-regulation	Ybx1,miR-214	Nfix induces Ybx1 degradation through ubiquitination, represses cyclin A2 and cyclin B1 expression, and inhibits cardiomyocyte proliferation. In addition, Nfix adsorbes miR-214, promotes the expression of Gsk3β, inhibites β-catenin activity, and arrests cardiomyocyte proliferation.

### Emerging key modulator in pre-existing cardiomyocyte proliferation

The proliferation of existing ACMs is considered as a potential source of new cardiomyocytes. Therefore, the formation of new cardiac myocytes after MI has important clinical significance for the treatment of MI. Recently, the proliferation of adult cardiomyocytes has made new progress. Employing β-Actin-green fluorescent protein transgenic mice and fate-mapping Myh6-MerCreMer-tdTomato/lacZ mice, the researchers tracked the fate and source of ACMs. An ischemia-resistant connexin 43 mutant enhances the Ca^2+^ signaling pathway of interstitial junctions in cardiomyocytes and forms new cardiomyocytes through dedifferentiation, proliferation, and re-differentiation of the cell cycle.^17^^,^^110^ In summary, emerging cell-cycle-associated modulators awaken adult cardiomyocytes to re-enter the cell cycle and trigger myocardial regeneration (Table 2).

**Table 2:** Emerging key modulator axis induce stable myocardial cell cycle re-entry and cardiac renewal.

Modulator	Ki67^+^ CM (%)	Model of cardiac injury	Regenerative signal axis
CHK1^111^	Rise 6.5% *in vitro* Rise 2.8% *in vivo*	AR in neonatal mice LAD ligation in adult mice	Targets mTORC1, activates P70S6K, and re-opens the regeneration window
HOXB13^112^	Rise 2.4% *in vivo*	LAD ligation in neonatal mice LAD ligation in adult mice	Meis1-Hoxb13 double-knockout displays CM mitosis, sarcomere disassembly and improved cardiac function.
Gp130^113^	Rise 1.9% *in vitro* Rise 4.1% *in vivo*	AR in adult mice LAD ligation in adult mice	Promotes cardiomyocyte proliferation by activating YAP via Src kinase
LRP6^114^	Rise 4.2% *in vitro* Rise 1.7% *in vivo*	LAD ligation in neonatal mice LAD ligation in adult mice	Regulates cardiomyocyte proliferation through the ING5/P21 pathway
PKM2^115^	Rise 3.4% *in vivo*	LAD ligation in adult mice	Interacts with β-catenin and activates both G6pd and the pentose phosphate pathway to provide nucleotides for DNA synthesis post-MI
ERBB2^18^	Rise 2.7% *in vitro* Rise 0.45% *in vivo*	LAD ligation in adult mice	ERBB2-ERK-YAP mechanotransduction involving EMT-like features was found to cause strong cardiac regeneration.
PITX2^38^	Unknown	AR in adult mice LAD ligation in adult mice	Pitx2-YAP interaction is found to be important for myocardial proliferation and regeneration.
MYDGF^45^	Rise 0.19% *in vitro* Rise 0.006% *in vivo*	AR in neonatal mice LAD ligation in adult mice	Activates c-MYC/FoxM1 pathway and goes against heart failure after cardiac injury
HIF1α^32^	Rise 0.003% *in vivo*	Chronic hypoxia exposure	Hypoxia exposure leads to cell cycle re-entry and cardiomyocyte expansion and induces myocardial regeneration in mammals.

In recent years, several emerging cell-cycle-associated functional proteins, such as CHK1, PKM2, HOXB13, and Gp130,^113^ have induced endogenous regenerative windows, thereby augmenting cardiomyocyte turnover and improving cardiac function. CHK1 is a serine/threonine protein kinase, an effector molecule for DNA damage checkpoints in the G2 phase. *In vitro*, CHK1 binds to and phosphorylates the bispecific protein phosphatases Cdc25A, Cdc25B, and Cdc25C, which dephosphorylate cyclin-dependent kinases to control cell cycle transitions.^116^^,^^117^ In the border zone of neonatal MI, quantitative proteomics, phosphorylation proteomics, and kinase-substrate network (KSN) analysis found the key kinase CHK1 for myocardial proliferation. Overexpression of CHK1 in the infarct border zone of adult mice can up-regulate the mTORC1/P70S6K pathway, promote cardiomyocyte proliferation, and improve cardiac function. This further proves that CHK1 triggers the regeneration of individual adult hearts by activating the mTORC1/P70S6K pathway.^111^ CHK1 is expected to become a potential new target for cardiomyocyte repair therapy after MI. In parallel, other recent studies have shown that the increase in oxidative stress in the early postnatal period is related to the transition from glycolysis to oxidative metabolism and plays a key role in cardiomyocyte cell cycle arrest.

Lineage tracing models show that transient PKM2 expression increases cardiomyocyte division and suppresses postnatal cardiomyocyte cell-cycle arrest. PKM2 is expressed in various cells, such as ESCs, adult stem cells, and especially tumor cells.^115^^,^^118^^,^^119^ Moreover, PKM2 directly interacts with β-catenin and up-regulates the anabolic enzymes G6pd and c-MYC, which reduces oxidative stress, ROS production, and oxidative damage and leads to increased expression of the cell cycle.^38^ So far, several regulators of postnatal cardiac cell cycle arrest have been reported, which highlights the complexity and necessity of research on the regulation of the cardiac cell cycle.^120^, ^121^, ^122^ HOXB13, as a cofactor of MEIS1 in cardiomyocytes, is a Ca^2+^ calmodulin-activated phosphatase, which cooperatively regulates the cell cycle and proliferation of cardiomyocytes, providing the research mechanism between the proliferation and hypertrophic growth of cardiomyocytes.^112^^,^^123^ LRP6 is a single transmembrane protein that regulates cell growth and mitosis in typical Wnt/LRP6 signaling and has been shown proved to affect the proliferation and survival of cardiac progenitor cells.^124^^,^^125^ Studies have shown that the synergistic effect of the ING5/p21 signaling pathway and LRP6 plays a key role in cardiomyocyte proliferation.^114^ The phosphorylation of YAP on S274 and S352 is necessary for mitosis and enhances the interaction of YAP with the cytoskeleton and nuclear envelope. To conclude, the researchers found a mechanical signal transduction pathway of ERBB2-ERK-YAP based on cytoskeleton remodeling and phosphorylation, which regulates EMT-like processes in cardiomyocyte mitosis and cardiac regeneration.^18^

### Extensive crosstalk of signaling pathways

**Figure 3:**
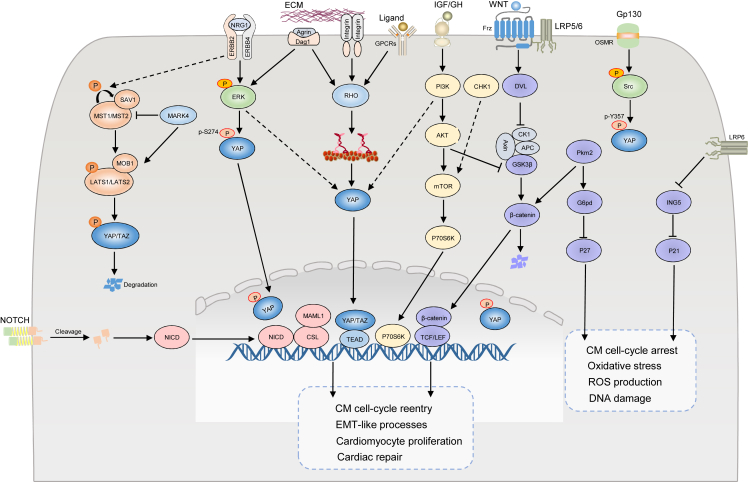
Mechanism of pre-existing myocardial proliferation and crosstalk with other emerging signaling pathways. Hippo-YAP, Notch, and Wnt/β-catenin pathway signaling, as a classic model of myocardial regeneration, plays a vital role in the proliferation after myocardial infarction (MI). Significantly, these signaling pathways do not exist independently but cooperate with other signaling molecules. For instance, NRG1/ERBB2, Wnt/β-catenin, PI3K/AKT/mTOR and Hippo-YAP signaling pathways cooperate in the regulation of robust cell-cycle activity and CM proliferation. In addition, some key signaling molecules, such as Pkm2, and LRP6, regulate the cardiomyocyte cell cycle and oxidative stress by inhibiting P21 and P27. In the prevention and therapy of MI, crosstalk between a wide range of signaling pathways triggers the re-entry of the myocardial cell cycle, which has ameliorated the amazing clinical application value of cardiovascular diseases.

Multiple studies have shown that these regeneration signaling pathways are not independent but form extensive crosstalk with other signaling pathways, leading to cardiomyocyte dedifferentiation and cardiac regeneration (Fig. 3). For instance, NRG1-ERBB2/ERBB4^26^ and Hippo-YAP signaling pathways cooperate to regulate robust cell-cycle activity and cardiomyocyte proliferation. It was found that in mice seven days postnatal, the endogenous overexpression of ERBB2 restricted GSK3β, enhanced the expression level of β-catenin, phosphorylated ERK, and AKT, and further activated the dedifferentiation markers RUNX1, cKIT, and DAB2. These core signaling molecules in the Hippo-YAP signaling pathway eenergetically push forward the progress of the NRG1-ERBB2/ERBB4 signaling pathway. In 2021, Dr. William T. Pu pointed out that PIK3CB is a critical direct target of YAP as a catalytic subunit of PI3K and a regulator of cardiac growth, which connects the Hippo-YAP and PI3K-AKT signaling pathways to promote the proliferation and survival of cardiomyocytes.^126^ In parallel, mTOR^127^ is responsible for important signal molecules in signal transduction and participates in regulating cell growth, survival, and metabolism via ribosomal protein S6 kinase β-1 molecules.

In the model of myocardial ischemia-reperfusion in mice, the forced overexpression of miR-496 can trigger the PI3K/Akt/mTOR signaling pathway, protect the heart from MI-induced apoptosis, and stimulate myocardial regeneration.^128^ Significantly, quantitative proteomics, phosphorylation proteomics, and KSN analysis have discovered the key kinase CHK1 for myocardial regeneration. After neonatal and adult cardiac MI, it has been shown that overexpression of CHK1 can improve the regeneration ability of cardiomyocytes of individual adult hearts by activating the mTORC1/P70S6K pathway.^111^ These findings establish an mTOR direct link between PI3K/Akt/mTOR and CHK1/mTORC1/P70S6K signaling pathways, which may be a potential network hub for myocardial repair therapy after MI. In the prevention and therapy of MI, crosstalk between a wide range of signaling pathways triggers the re-entry of the myocardial cell cycle, which has ameliorated the significant clinical application value of heart injury repair and cardiovascular disease treatment.

## Conclusion

After more than a decade of intense research, cardiac regeneration science appears to be catching up. Fundamental activation of the cardiomyocyte cycle and re-entry, and then extending the 7-day window period of cardiomyocyte proliferation is a key step for endogenous myocardial regeneration.^111^ Different animal models have revealed numerous regulatory factors controlling the molecular mechanisms of cardiac regeneration. These mechanisms include epicardial signaling, immune responses, ECM, and neuromodulation. Several specific ligands promote cardiomyocyte proliferation and cardiac regeneration, including NRG1, fibroblast growth factor 1, Wnt, and tumor necrosis factor.^129^ Different physiological and pathological environments, such as inflammatory factors, hypoxia, and iron metabolism, actively participate in the process of changing the fate and regeneration of cardiomyocytes. The loss or gain of some regulatory factors controls the re-entry of the adult myocardial cell cycle, thereby reducing the level of fibrosis and weakening the response of myocardial dysfunction to MI. A series of acting factors may be key signals that trigger the proliferation of pre-existing cardiomyocytes in the heart. Endogenous key cardiomyocyte proliferation regulators, such as cell cycle-related emerging proteins and CDKs, promote stable cell cycle re-entry.

Cell cycle-related novel proteins and ncRNAs promote endogenous cardiac regeneration by inducing pre-existing cardiomyocyte proliferation in mice. These emerging and budding studies have aroused great interest in exploring cardiac regeneration. Transient signal clusters in adult mouse cardiomyocytes form the main crosstalk pattern to activate cardiac regeneration, leading to the formation of new cardiomyocytes. These signal transduction involving non-classical regenerative pathways constitute a relatively sound network of regulatory homeostasis. Although a large number of current studies support the opening of a window for the development of effective cardiovascular drug therapies, effective and specific delivery to the target is a fundamental problem that must be addressed. Recently, the development of small molecules to induce cardiac regeneration in adult mammals has shown initial success. A chemical cocktail of five small molecules triggers the lactate-LacRS2-mTOR signaling, leading to CM metabolic switching toward glycolysis/biosynthesis and CM de-differentiation before entering the cell cycle.^130^ Mature bioengineered collagen patches were used as scaffolds for delivering recombinant RELN protein into the heart.^131^ The size of the fibrotic scar was notably reduced in RELN-patched mice. In this way, we can assume that the induction of quiescent CMs into cell division and cardiac regeneration requires the simultaneous intervention of multiple signaling pathways.

However, we still do not know enough about the mechanisms of cardiac regeneration. Cardiac regeneration requires a complete system including nerve and vascular reconstruction, triggering various signaling pathways, and synergistically completing the balance of cardiac development and homeostasis. Thus, exploring the mechanisms of adult pre-existing cardiomyocyte proliferation and therapeutic targets for cardiac repair has become a top priority in cardiovascular research.

## Author contributions

T.W. and X.C. reviewed the literature and drafted the manuscript; K.W., J.J., X.Y., and W.Y. designed the figures; C.L. and Y.W. provided supervision and revised the manuscript. All authors approved the final manuscript.

## Conflict of interests

The authors declare no conflict of interests.

## Funding

This work was supported by the 10.13039/501100001809National Natural Science Foundation of China (No. 82070314, 81600244) and the 10.13039/501100007129Natural Science Foundation of Shandong Province, China (No. ZR2021MC189).
